# Polymer Microfibers Incorporated with Silver Nanoparticles: a New Platform for Optical Sensing

**DOI:** 10.1186/s11671-019-3108-6

**Published:** 2019-08-08

**Authors:** Muhammad Khuram Shahzad, Yundong Zhang, Adil Raza, Muhammad Ikram, Kaiyue Qi, Muhammad Usman Khan, Muhammad Jehanzaib Aslam, Abdulaziz Alhazaa

**Affiliations:** 1 National Key Laboratory of Tunable Laser Technology, Institute of Opto-Electronics, Department of Electronic Science and Technology, Harbin Institute of Technology (HIT), Harbin, 150080 People’s Republic of China; 2 College of Material Science and Technology, Nanjing University of Aeronautics and Astronautics, 29 Yudao Street, Nanjing, 210016 People’s Republic of China; 3 Solar Cell Applications Research Lab, Department of Physics, Government College University, Lahore, Punjab 54000 Pakistan; 4 Research Chair for Tribology, Surface, and Interface Sciences, Department of Physics and Astronomy, College of Science, King Saud University, Riyadh, Saudi Arabia; 5 King Abdullah Institute for Nanotechnology, King Saud University, Riyadh, Saudi Arabia

**Keywords:** Microfibers, Up-conversion luminescence, Er^3+^, Ag, Transmission losses, Sensitivity

## Abstract

The enhanced sensitivity of up-conversion luminescence is imperative for the application of up-conversion nanoparticles (UCNPs). In this study, microfibers were fabricated after co-doping UCNPs with polymethylmethacrylate (PMMA) and silver (Ag) solutions. Transmission losses and sensitivities of UCNPs (tetrogonal-LiYF_4_:Yb^3+^/Er^3+^) in the presence and absence of Ag were investigated. Sensitivity of up-conversion luminescence with Ag (LiYF_4_:Yb^3+^/Er^3+^/Ag) is 0.0095 K^−1^ and reduced to (LiYF_4_:Yb^3+^/Er^3+^) 0.0065 K^−1^ without Ag at 303 K under laser source (980 nm). The UCNP microfibers with Ag showed lower transmission losses and higher sensitivity than without Ag and could serve as promising candidate for optical applications. This is the first observation of Ag-doped microfiber via facile method.

## Background

Up-conversion nanoparticles (UCNPs) after co-doping with lanthanides ions have drawn much attention due to application in imaging, laser materials, display technologies, and solar cells [1–3]. The low fluorescence emission efficiency of UCNPs can be caused by the small absorption coefficients of lanthanide ions. The nanoscale dispersion of metal nanoparticles in polymeric and inorganic substrates has triggered a great interest in novel physical, chemical, and biologic properties of the nanocomposite materials [4]. For potential applications of the further miniaturization of electronic components, optical detectors, chemical and biochemical sensors, and devices are exciting possibilities with metal nanoparticles. Additionally, the semiconductors have been used as sensitizers for widening absorption range, such as CdSe, CdS, PbS, WO_3_, and Cu_2_O [5, 6]. Among these semiconductors, Cu_2_O is an interesting candidate due to its narrow band gap of ~ 2.1 eV, non-toxicity, low cost and abundance but heterostructure of Cu_2_O/ZnO is a promising material structure. It leads to a functional intergration, novel interface effect's properties of Cu_2_O and ZnO material [7]. On the other hand, UCNPs depicts superior properties relative to semiconductor quantum dots for instance the absence of autofluorescence tissue penetrability near-infrared laser excitation, non-blinking, and high chemical stability [8]. The synthesis of lanthanide-doped materials with spherical nanoparticles and nanorods has been studied by many research groups [9]. The issue of UCNPs oxidation occurs at high temperature significantly which reduced their applications. To avoid oxidation, core/shell structure overcomes oxidation whereas SiO_2_ shell grows around nanocrystals. Nanocrystal integration on chip as microstructure light detector is difficult. Therefore, microtubes, quantum dot-doped nanofibers, and dye-doped polymer nanowires have been employed in microstructural optoelectronics technology after successful investigation [10]. Correspondingly, nanowires, microtubes, and nanofibers have been fabricated and utilized to discuss the thermal sensing behavior by different research groups [11, 12].

However, metal nanoparticles (MNPs) have been considered to enhance UCNPs efficiency. Different strategies including chemical modification, crystal structure, and local field adjustment of metal have been proposed to improve the efficiency and sensitivity [13]. Investigations on rare earth ion-doped luminescence materials for luminescence enhancement of metal nanostructure such as Er^3+^/Yb^3+^ co-doped bismuth-germinate glasses containing Ag nanoparticles and Er^3+^/Yb^3+^ co-doped β-NaLuF_4_ nanocrystals which are spin-coated over gold NPs have been reported with inconsistent results and high sensitivity [14]. Moreover, aggregation-induced emission (AIE) is a distinctive fluorescence phenomenon which suggested that few dyes can emit stronger fluorescence in their solid state than in dispersion solution [15–17]. Different mechanism including J-aggregate formation, conformational planarization, and twisted intramolecular charge transfer for the AIE phenomenon has been previously proposed by researchers [18–22]. Besides, materials with AIE characteristics have attracted more research attention for potential application in various field organic light-emitting diode, chemosensing, and bioimaging [23–27]. Especially, the preparation of AIE-active fluorescent organic nanoparticles has attracted attention recently. These materials containing AIE dyes could emit strong luminescence in physiological solution which effectively conquers the aggregation-caused quenching effect of fluorescent organic nanoparticles based on typical organic dyes [28, 29]. Although many strategies for the preparation of AIE-active fluorescent organic nanoparticles have been developed, the preparation of AIE-active through facile and effective multicomponent reaction (MCR) has received rarely attention due to mismatch with experimental data [30–34]. So, the unique AIE properties of dyes showed very promising for the fabrication of ultra-bright luminescent polymeric nanoparticles [35, 36].

In maximum experimental study, powder samples were used to perform the spectral measurements that increased the concerns regarding the influence of aggregation inter-reflection. Therefore, it is necessary to establish a facile and simple strategy to overcome the abovementioned drawbacks. Thus, Ag nanoparticles after co-doping with UCNPs and PMMA solution were used in microfibers to enhance the luminescence. However, no results have been described focusing on Ag co-doped UCNPs to microfibers (UCNPs-MF).

Herein, we present a facile method to prepare microfibers from UCNPs/PMMA with and without Ag solutions. Especially, the photoluminescence properties of Ag and absence of Ag co-doped microfibers are studied at various excitation point of microfibers. Moreover, UC luminescence characteristics of a microfiber is investigated by exciting 980 nm diode laser source at different temperature for the purpose of temperature sensing. The dependence of the integrated FIR on temperature is obtained and the experimental data can be fitted well with an exponential function. Thus, a single microfiber having transitions 2H11/2→4I15/2 and 4S3/2→4I15/2 levels at 522 and 541 nm is used to calculate the thermal sensitivities.

## Experimental and Method Section

### Materials

The silver (Ag) powder, chloroform, cyclohexane, NaOH, NH_4_F, and ethanol were purchased from Shanghai Chemical Company, China. These chemicals were of analytic grade and used without further purification.

### Preparation of Tetrogonal-LiYF_4_: Yb^3+^/Er^3+^ Nanoparticles

UCNP (tetrogonal-LiYF_4_:Yb^3+^/Er^3+^) was prepared using thermal decomposition technique. The three-necked flasks of 100 mL were used which contain rare earth ions with LnCl_3_ (Ln=Lu, Yb, Er) having a molar ratio of 78:22:1, respectively. The solution includes 15 mL 1-octadecene (ODE) and 6 mL oleic acid (OA). The mixture was heated up to 150 °C to obtain a pellucid solution and cooled up to room temperature after eliminating oxygen and residual water. Four millimoles of NH_4_F and 2.5 mmol of NaOH were added slowly into a flask containing 10 mL solution of methanol. To confirm, fluoride was dissolved entirely by stirring process up to 30 min after that prepared solution was heated at 300 °C at a rate of 50 °C/min for 1 h under argon atmosphere. The precipitates were separated at the rate of 4000 rpm and cooled down to room temperature, washed with ethanol, and dried at 60 °C for 12 h.

### Fabrication of Ag Co-doped Fibers

**Fig. 1:**
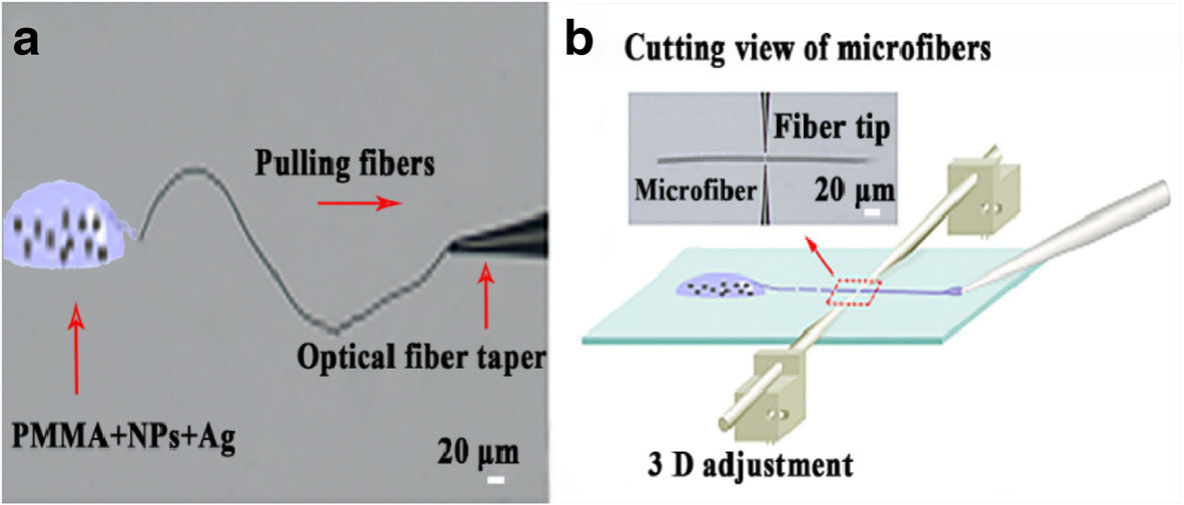
Fabrication process of Ag co-doped microfibers (**a**) Pulling of microfibers from PMMA+NPs+Ag solutions. **b** Cutting view of fabricated microfibers into small pieces

In a typical fabrication process, 0.003 g of Ag, 0.005 g of tetrogonol-LiYF_4_:22%Yb^3+^/1%Er^3+^, and 0.6 g of PMMA were mixed separately in 15 ml, 12 ml, and 18 ml of cyclohexane (C_6_H_12_) and chloroform (CHCl_3_) solution, respectively. Afterwards, the mixture of PMMA gradually dispensed into Ag and UCNP solutions and stirred for 30 min until a transparent solution was obtained. A fiber probe with a tip several microns in size was fabricated using the flame-heated drawing technique. After the mixed solution was dropped on the glass substrate, a fiber probe was then dipped into the mixed solution and withdrawn rapidly to fabricate the microfibers. The microfibers were then drawn and cut into small pieces, as shown in Fig. 1.

### Spectra Measurement

**Fig. 2:**
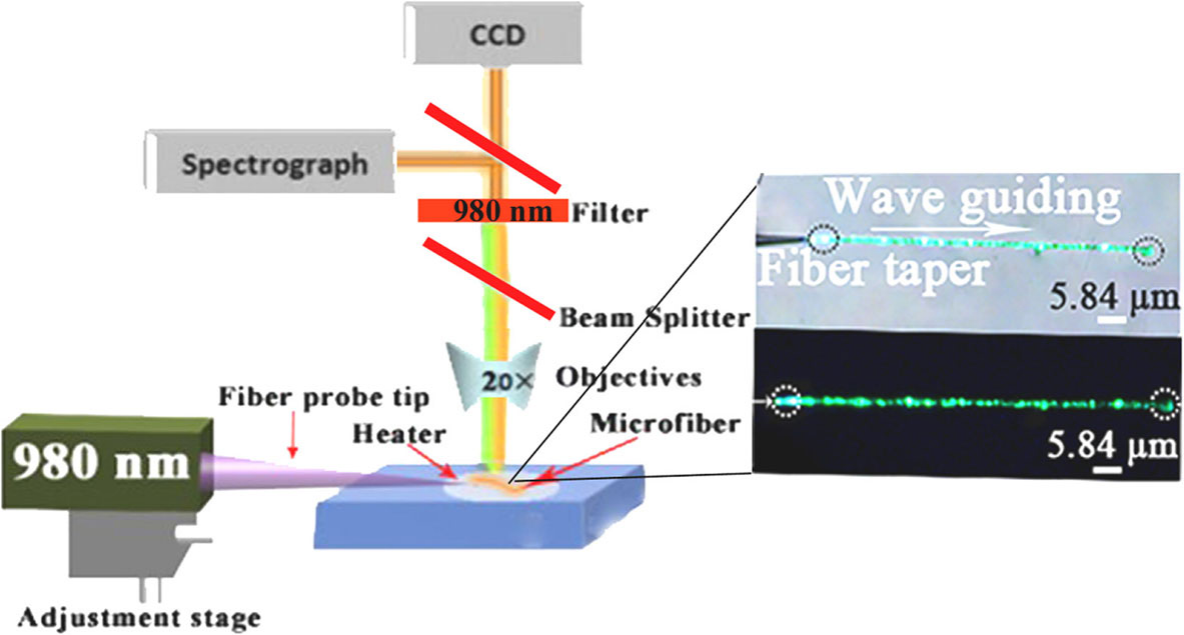
Experimental setup of wave-guiding phenomena

Figure 2 demonstrates the experimental setup, to study the thermal and optical properties of microfibers. The microfibers were illuminated using an excitation source of 980 nm after depositing on a glass substrate. In order to measure the transmission losses of microfibers, ×20 objective (NA = 0.4) was used. The charge-coupled device (CCD, ACTON) camera was applied to obtain emission spectra of a microfiber, and ocean optics spectrometer was used to record the spectra for temperature-sensing measurement. The excitation of microfibers having different diameter was demonstrated with 980 nm laser source under 0.998 mW laser power to study microscopic thermal properties.

## Results and Discussion

### Structure and Transmission Properties

**Fig. 3:**
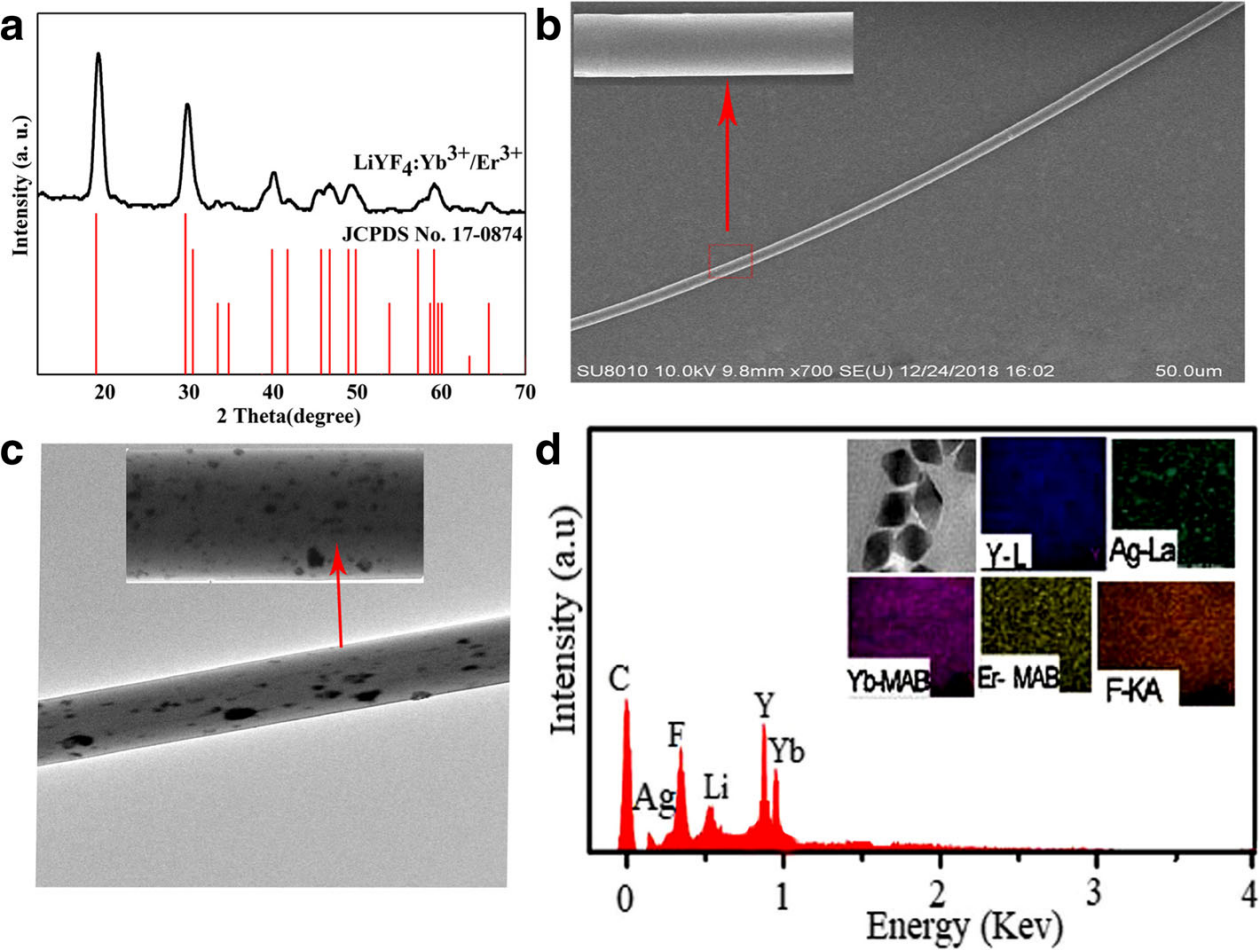
Characterization process of LiYF_4_:Yb^3+^/Er^3+^ and Ag co-doped microfibers. **a** XRD of LiYF_4_:Yb^3+^/Er^3+^. **b** SEM of Ag co-doped microfiber. **c** TEM Of Ag co-doped microfiber. **d** EDS of Ag co-doped microfiber

Phase purity and crystal structure of UCNPs were studied by applying X-ray diffraction (XRD, Rigaku Miniflex II) technique. The observed XRD peak patterns (Fig. 3a) are well indexed and in agreement with JCPDS card # 17-0874. Fig. 3(b) displays scanning electron microscopy (SEM, NOva Nano-SEM 650) images of a microfiber. One of the SEM image could be clearly seen (see the inset) which suggests that a microfiber has a uniform diameter, together with a smooth surface. For better resolution, we used transmission electron microscopy (TEM, Tecnai G2F30) and energy-dispersive X-ray analysis (EDS, Tecnai G2F30) to investigate individual Ag co-doped microfibers. Figure 3(c, d) shows TEM and EDS images, respectively, which confirm the strong evidence of uniform dispersion of Ag co-doped nanoparticles in a single microfiber.

**Fig. 4:**
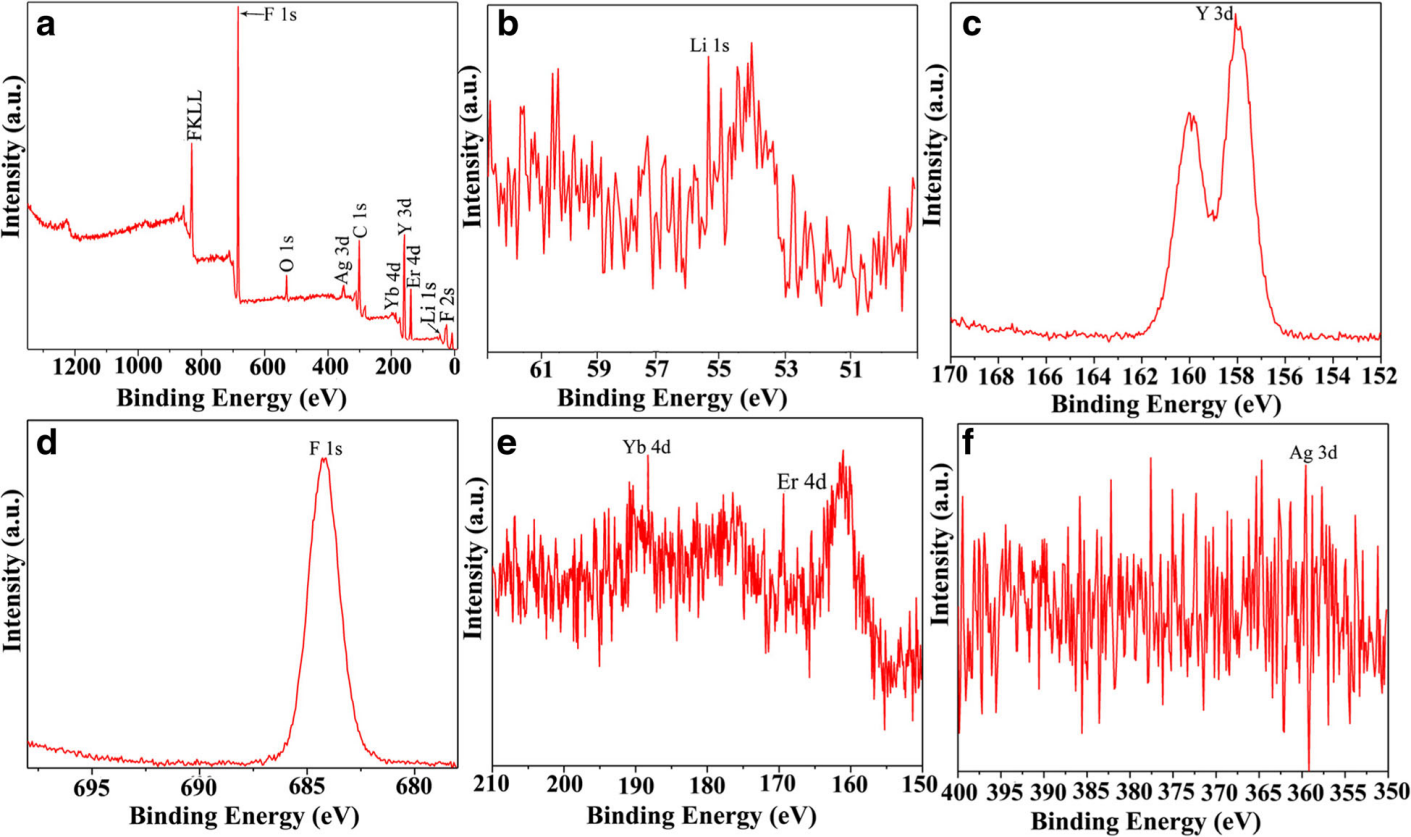
XPS **a** survey, **b** Li 1s, **c** Y 3d, **d** F 1s, **e** Yb and Er 4d, and **f** Ag 3d spectra of LiYF_4_:Yb^3+^/Er^3+^ NPs doped with Ag

Furthermore, X-ray photoelectron spectroscopy (XPS, Thermofisher Escalab 250Xi) was used to determine the successful incorporation of rare earth ions and Ag ions into the LiYF_4_ host material as shown in Fig. 4a–f. The XPS survey spectrum (Fig. 4a) shows the presence of Li, Y, F, Yb, Er, and Ag elements, and the peak at 55.25 eV can be assigned to the binding energy of Li 1s (Fig. 4b). The peaks observed at 158.08 eV (Fig. 4c) can be assigned to the Y 3d. The peak at 684.08 eV is attributed to the binding energy of F1s (Fig. 4d). The Yb 4d and Er 4d peaks (Fig. 4e) can be observed at 186.08 and 164.08 eV, respectively. The peak located at 359.08 eV is related to the binding energy of Ag 3d. This confirms the successful tridoping of Ag ions in LiYF_4_:Yb^3+^/Er^3+^ nanoparticles [37].

**Fig. 5:**
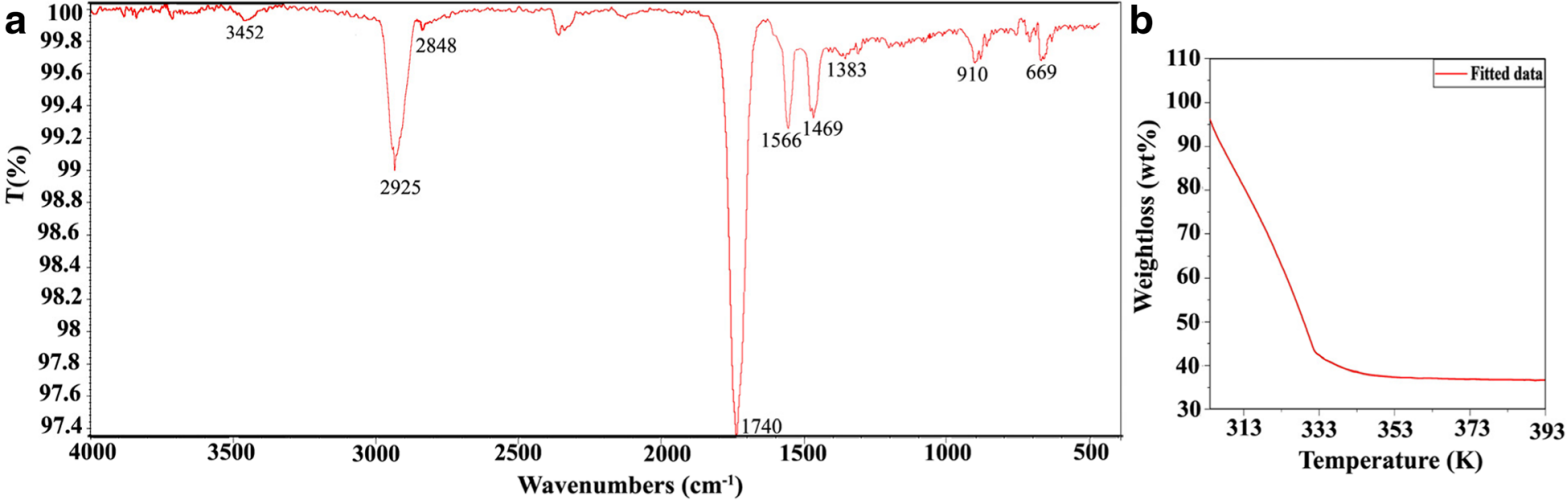
**a** FTIR spectra of LiYF_4_: Er^3+^/Yb^3+^/Ag. **b** TGA spectra LiYF_4_: Er^3+^/Yb^3+^/Ag

Figure 5a shows Fourier transform infrared ray (FTIR, Nicolet50 NTA449F3) spectra of LiYF_4_:Yb^3+^/Er^3+^/Ag nanoparticles in the region 400–4000 cm^−1^. The studies were carried out in order to ascertain the purity and nature of nanoparticles. The peaks observed at 3452 cm^−1^ are may be due to O-H stretching and deformation. The bands at 2925 and 2848 cm^−1^ are associated to the asymmetric (u_as_) and symmetric (u_s_) stretching vibration of methylene (−CH_2_) in the long alkyl of oleate molecule, respectively. The bands at 1566 and 1469 cm^−1^ can be assigned to the asymmetric (u_as_) and symmetric (u_s_) stretching vibration of the carboxylic group, respectively. The spectra contain a peak at 1740 cm^−1^ due to C=O stretching vibration. The peak located at 1383 cm^−1^ corresponds to the C-H deformation vibration. The spectra also contain a peak at 910 and 669 cm^−1^ which is due to asymmetric stretching vibration and Ag-O deformation vibrations. It implies that FTIR results are in accordance with literature values [38].

To better understand the formation mechanism of Ag-doped microfibers, the thermal gravimetric analysis (TGA, NETZSCH) was conducted under a dry airflow from 293–393 K temperature. It is observed in Fig. 5b that a microfiber shows roughly two degradation steps. The first weight loss below 333 K could be attributed to loss of absorbed moisture/with the evaporation of trapped solvent (H_2_O or CHCl_3_) which is independent of sample composition. In graph, second weight loss happens from 333 K to 393 K which clearly represents the polymeric degradation process. Hence, Ag co-doped microfibers are polymer-based fibers which cannot stand with the temperature above 332 K [4].

**Fig. 6:**
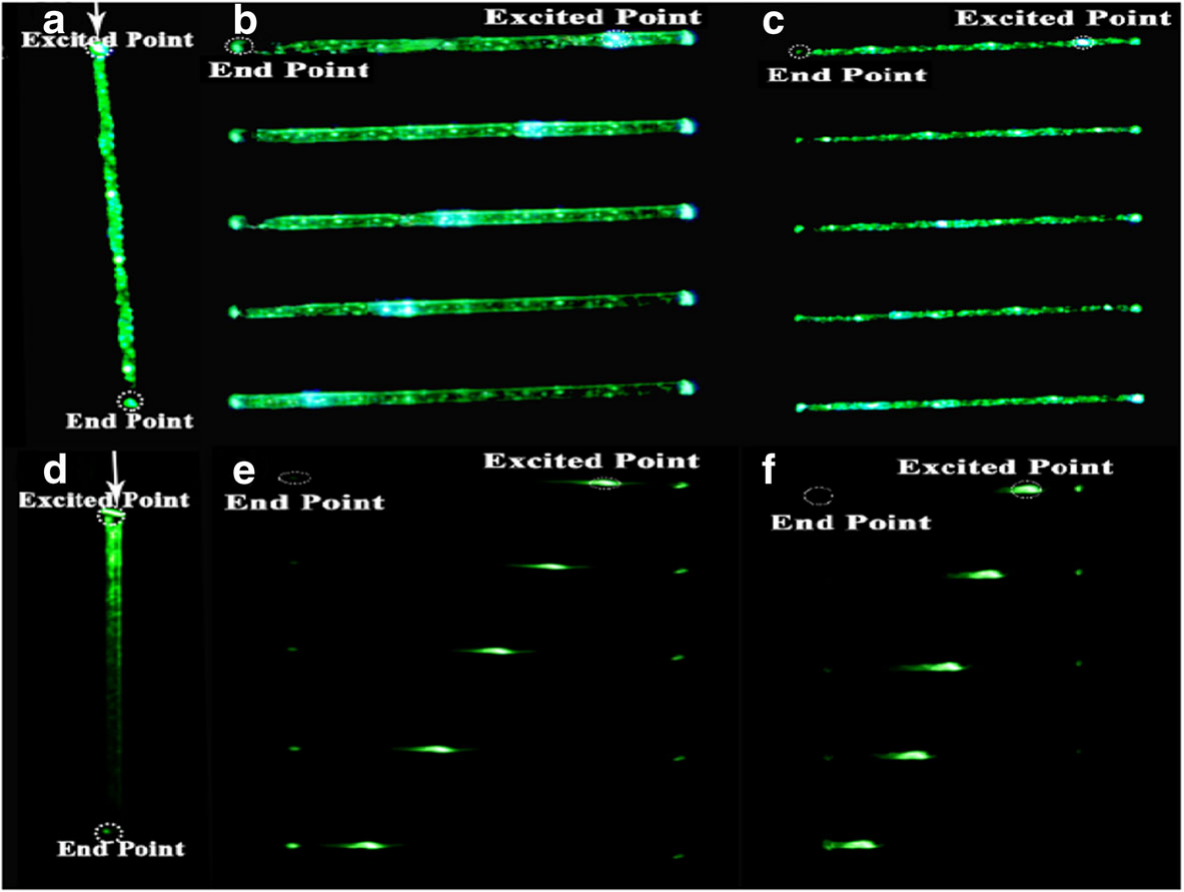
Photoluminescence images with different diameter of microfibers. **a**–**c** Luminescence of Ag microfiber under dark background. **d**–**f** Excitation without Ag microfiber under black background

In order to investigate individual optical properties of Ag-doped and undoped microfibers, laser light (980 nm) was employed from standard optical fiber to expose microfibers at oblique angles with respect to microfibers along axis. Figure 6a shows Ag co-doped microfiber (diameter ~ 6 μm) which was vertically excited under dark background with 980 nm and appeared that light spread in whole fiber because of Ag co-doped nanoparticles served as light transmitter. Conversely, Fig. 6d depicts without Ag co-doped microfiber (diameter ~ 6.5 μm) which was excited under dark background at top position with 980 nm laser source. It suggests that light cannot transmit equally in fiber due to high self-absorption and Rayleigh scattering phenomena. A microfiber (diameter ~ 6 μm) containing Ag co-doped NPs shows high green light emission than undoped Ag (diameter ~ 6.5 μm) having the same excitation of laser source under dark field. It is observe that bright end spots with no cluster having optical waveguides intend Ag co-doped microfiber absorbs near IR light and conduct alike toward end points. Moreover, Fig. 6b and c indicate that the Ag co-doped fibers having different diameters (~ 15.55 and ~ 9.15 μm) were excited at five different positions and exhibited green light emissions toward end points. Conversely, 980 nm laser source was applied to excite microfibers (without Ag NPs) at different five position having different diameters (~ 11.89 and 14.57 μm) which are shown in Fig. 6e–f, indicating less green light emission toward end points. The photoluminescence (PL) intensity of excited points against end spots was performed to elaborate the wave-guiding performance of microfibers (with and without Ag NPs) quantitatively [39]. We used adobe photoshop to convert spot images from RGB to gray styles, these gray values were evaluated by using MATLAB to characterize the corresponding intensities. After normalizing end points of photoluminescence intensities toward excited points, decay curves dependent of light propagation distance were obtained.

The transmission losses were measured using equation [40]:

1 $$ \frac{I_{\mathrm{endpoint}}}{I_{\mathrm{O}}}=\exp \left(-\upalpha \mathrm{d}\right) $$

**Fig. 7:**
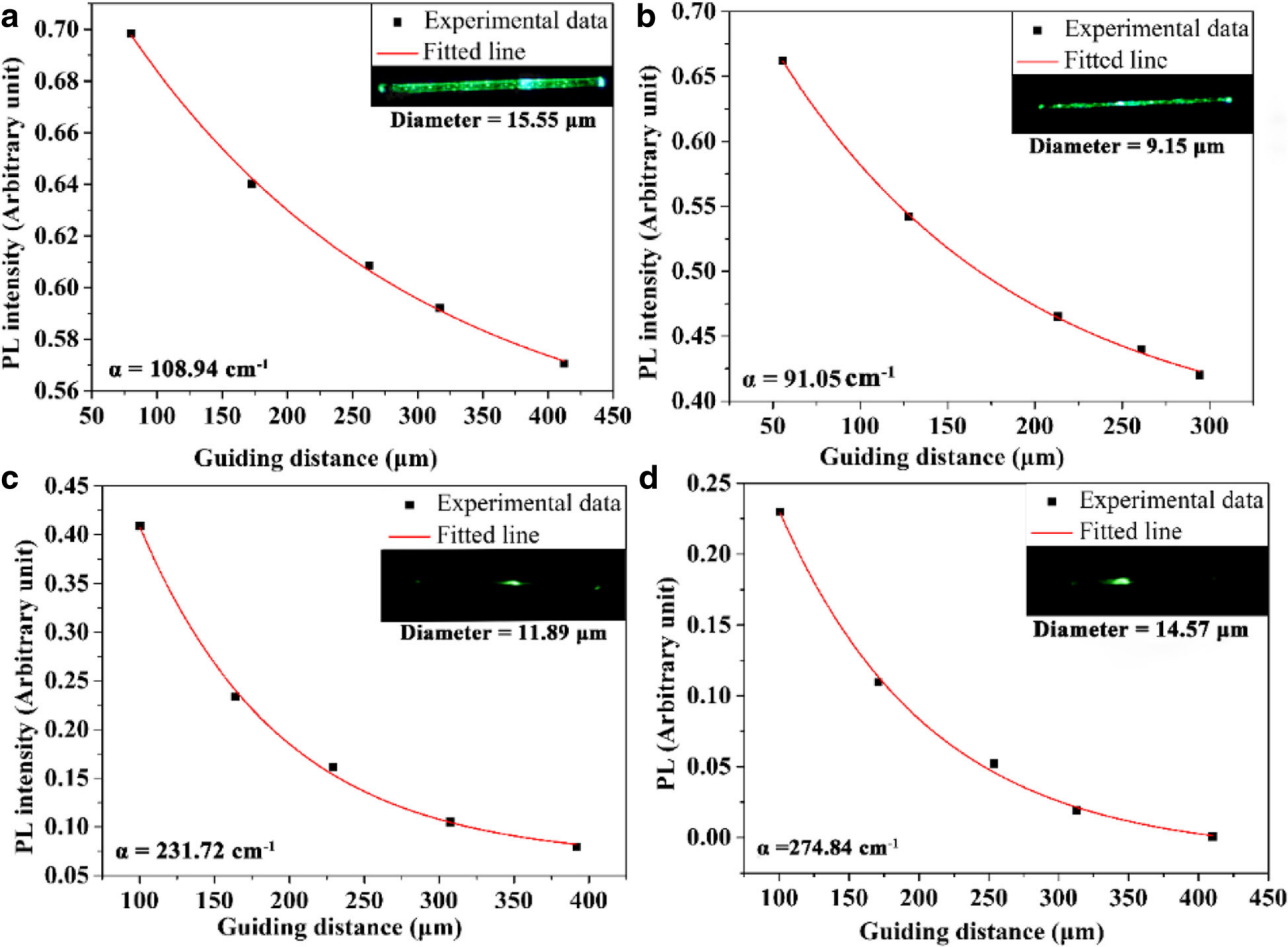
**a**, **b** Fitting lines between photoluminescence (PL) intensity and guiding distance of different diameter of microfibers with Ag co-doped under different excitation point. **c**–**d** Fitting lines between photoluminescence (PL) intensity and guiding distance of different diameter of microfibers without Ag co-doped under different excitation point

Here, Eq. (1) shows that excited spots distance increases which results exponentially decrease of photoluminescence intensity. The relationship between photoluminescence intensity as a function of guiding distance of fibers (~ 15.55 and ~ 9.15 μm) with Ag NPs is shown in Fig. 7a, b. The emitted spectra were collected at five positions along the axis of microfibers which specifies the transmission of laser light with transmission loss coefficients α = 108.94 cm^−1^ and 91.05 cm^−1^. Conversely, Fig. 7c, d demonstrates the transmission loss coefficients of microfibers (without Ag NPs) having a diameter of 11.89 and 14.57 μm are about 231.72 and 274.84 cm^−1^, respectively. It is noteworthy that when the light is guided through Ag co-doped microfibers, it maintains small mode areas along the entire length of fiber. It enables strong interaction between light and Ag nanoparticles in cascade and leading to light transfer with high efficiency relative to microfibers without Ag. Ag co-doped nanoparticles have highly efficient photon to plasmon conversion in wave-guiding microfibers and facilitated enhanced light matter interactions within a highly localized area [41]. It accelerates opportunities for developing Ag-based photonic components and devices having high compactness, low optical power consumption, and reduced sizes. It is noted that simultaneous multiphoton excitation has been widely applied in fluorescent optical microscopy to show increased resolution and decreased specimen autofluorescence, as well as increased imaging depth. However, the low NIR absorption cross-section of multiphoton labels requires this technique to subject to the use of high-peak power ultrashort-pulsed laser. Principally distinct from simultaneous multiphoton process in dyes and QDs, which involves the use of a virtual energy level, photon up-conversion in UCNPs relies on the sequential absorption of low energy photons through the use of ladder-like energy levels of lanthanide doping ions. This quantum mechanical difference makes UCNP orders of magnitude more efficient than multiphoton process, allowing excitation with a low-cost continuous-wave laser diode at low-energy irradiance, typically as low as ∼ 10^−1^ W.cm^−2^ [42]. The microfibers (UCNPs/PMMA/Ag) possess favorable transmission properties. Thus, the proposed microfibers (UCNPs/PMMA/Ag) have advantages of easy fabrication, low cost, strong plasticity, and unique optical properties of UCNPs such as large anti-stokes shift and abundant emission bands, further supporting their applications based on optical signal transmission, sensors, and optical components. Consequently, our estimated results of wave-guiding performances show well agreement with reported work [43, 44].

### Energy Levels and Thermal Effects

**Fig. 8:**
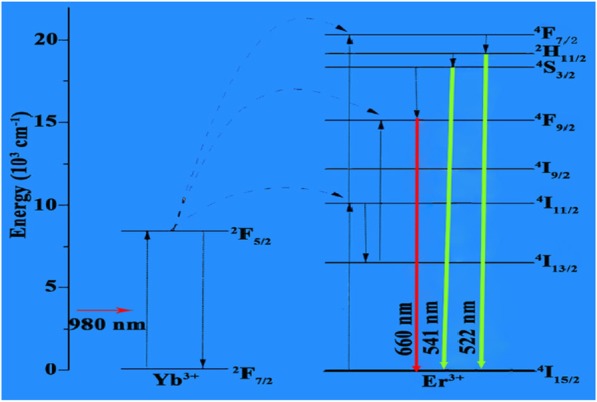
Energy level diagram of LiYF_4_:Yb^3+^/Er^3+^

To elaborate the energy level diagram of UCNPs (Yb^3+^/Er^3+^), two dominant green emission bands around 522 and 541 and a red emission band centered at ~ 660 nm were observed. These observed emission lines are originated from ^2^H_11/2_ → ^4^I_15/2_, ^4^S_3/2_ → ^4^F_9/2_, and ^4^S_3/2_ → ^4^I_15/2_ of Er^3+^ ions, respectively. Energy levels ^2^H_11/2_ and ^4^S_3/2_ are populated by two photon processes. For population system of Yb^3+^/Er^3+^ ions, Yb^3+^ ions are excited by the pumping photons to populate consecutive three levels of Er^3+^ ions which are demonstrated as ^4^I_11/2_, ^4^F_9/2_, and ^2^H_11/2_ levels. It is observed that the population of ^2^H_11/2_ is obtained from given process ^4^I_15/2_ → ^4^I_11/2_ (Er^3+^): ^4^I_11/2_ → ^2^H_11/2_ (Er^3+^) levels. This phenomenon is caused by temperature excitation between thermally coupled levels. Therefore, the populations of ^2^H_11/2_ and ^4^S_3/2_ satisfy the Boltzmann statistics resulting in variation of population rates of ^2^H_11/2_ → ^4^I_15/2_ and ^4^S_3/2_ → ^4^I_15/2_ levels [45]. The mechanism of up-conversion process in Er^3+^/Yb^3+^ is illustrated in Fig. 8.

**Fig. 9:**
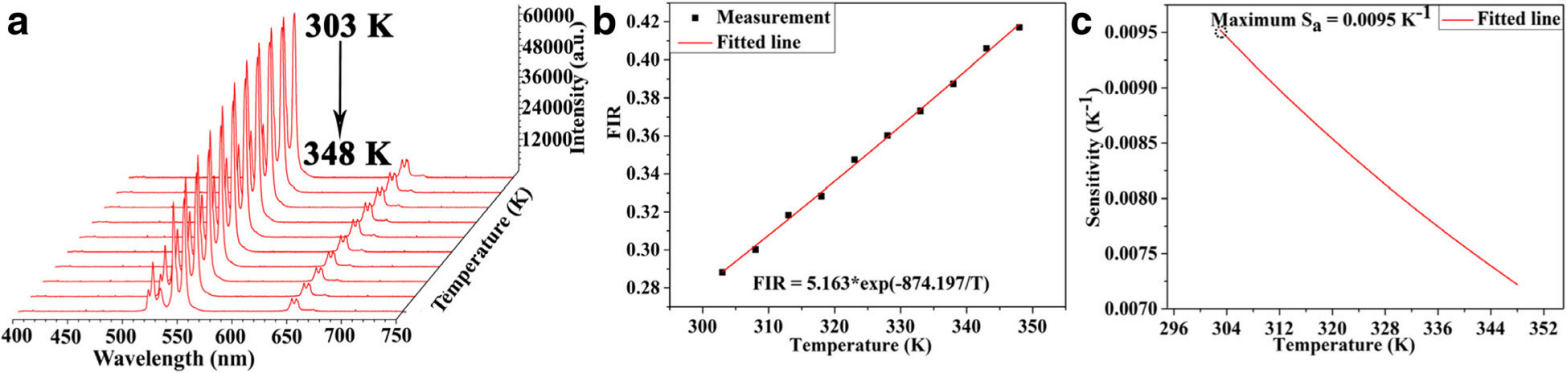
**a** 3D up-conversion emission spectra of Ag co-doped microfiber under 980 nm excitation source. **b** Fitted curves between fluorescence intensity ratio and temperature. **c** Fitted data between sensitivity (K^−1^) and temperature (K) of Ag co-doped microfiber

**Fig. 10:**
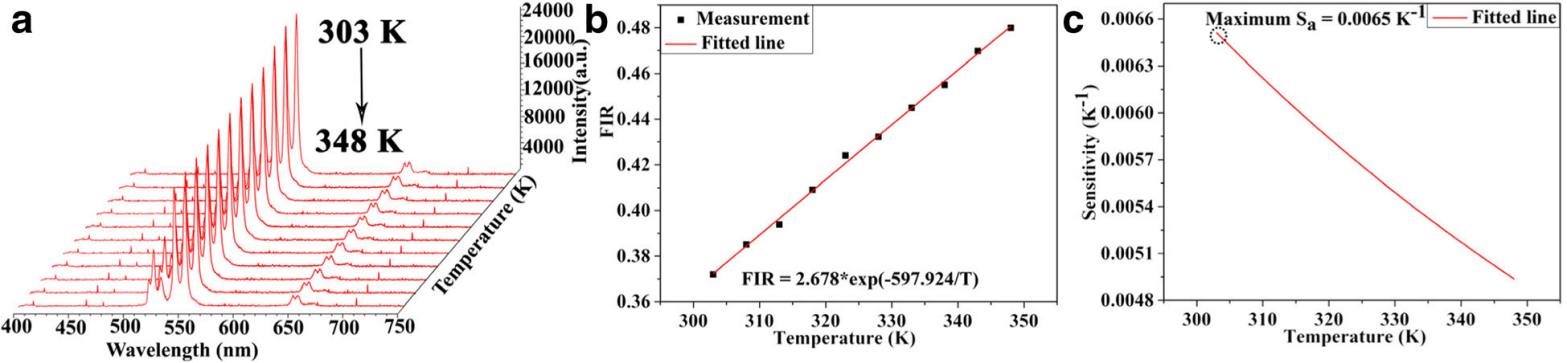
**a** 3D up-conversion emission spectra without Ag under 980 nm excitation source. **b** Fitted curves between fluorescence intensity ratio and temperature without Ag. **c** Fitted data between sensitivity (K^−1^) and temperature (K) without Ag

The Ag co-doped UCNPs in fibers showed spectra with 980 nm laser source. The up-conversion (UC) luminescence is suitable for temperature-sensing applications. Therefore, Figs. 9a and 10a depicted the emission spectra of Ag and without Ag co-doped NPs which ranged from 400 to 750 nm under fiber laser excitation source, and spectra were collected with an average increment of 5 °C in temperature regime (303–348 K). Interestingly, by increasing temperature, the emission intensities were decreased significantly, therefore using 0.998 mW laser powers to avoid from thermal effects, clearly indicating the temperature-dependent behavior. While UCNPs-MF was heated in the temperature domain of 348–303 K, all photoluminescence was restored to original position whereas intensities showed significant reduction upon increasing the temperature. Therefore, this significant reduction in intensity is attributed to the escalation of variety of relative intensity corresponding to several multiphonon relaxation rates to diverse multiphonon relaxation rate. The luminescent intensity is significantly increased by introducing Ag in a microfiber under same experimental condition. Typically, heat energy is generated by laser light near irradiated area whose temperature is measured by applying thermal sensors, to estimate temperature of irradiated point with great accuracy. Fluorescence intensity ratio technique is a versatile technique widely used for temperature estimation. We discussed Ag and without Ag co-doped fibers upon temperature fluctuation; populations of ^2^H_11/2_ and ^4^S_3/2_ followed the Boltzmann distribution which resulted in variable population rates of ^2^H_11/2_ → ^4^I_15/2_ and ^4^S_3/2_ → ^4^I_15/2_. Temperature sensing can be calculated using intensity ratio between ^2^H_11/2_ → ^4^I_15/2_ and ^4^S_3/2_ → ^4^I_15/2_ transitions. Fluorescence intensity ratio (FIR) method can be expressed from the following equation [46]:

2 $$ \mathrm{FIR}=\frac{I_{522\mathrm{nm}\kern0.75em }}{I_{541\mathrm{nm}}}=C\exp \left(-\frac{\Delta E}{kT}\ \right) $$

Here, *I*_522nm_ and *I*_541nm_ are the relative intensities, *C* is the proportionality constant, ΔE is the energy gap between 522 and 540 nm, T is the absolute temperature, and *k* is the Boltzmann constant. Moreover, Figs. 9b and 10b display the variation of FIR with temperature; Eq. (2) determined that observed experimental data have a good linear fitting relationship. It is worth to investigate another key parameter that is the thermal-sensing mechanism of Ag- and without Ag-doped microfiber. Therefore, sensitivity (*S*) can be written as follows [47]:

3 $$ {S}_{\mathrm{a}}=\frac{\mathrm{FIR}}{\mathrm{dT}}=\mathrm{FIR}\left(\frac{\Delta E}{kT^2}\right) $$

Here, *S*_a_ is the absolute sensitivity of Ag and without Ag co-doped microfibers. The curves are exhibited in Figs. 9c and 10c, but digital values (FIR, *ΔE*, and *k*) for Ag and without Ag are obtained by fitted curves presented in Figs. 9b and 10b. Maximum sensor sensitivities for LiYF_4_:Yb^3+^/Er^3+^ and LiYF_4_:Yb^3+^/Er^3+^/Ag demonstrated to be 0.0065 and 0.0095 K^°1^ at 303 K, respectively. The optical temperature sensor’s sensitivities in different host materials are listed in Table 1. Although other sensitivities have a higher value as compared to without Ag UCNPs, LiYF_4_:Yb^3+^/Er^3+^/Ag is superior to host materials.

**Table 1:** The sensitivity values of optical temperature sensors in different host materials

Materials	Temperature (K)	Transitions	Intensity-dependent temperature sensitivity (K^-1^)	Ref.
Er-Mo:Yb_2_Ti_2_O_7_	295–973	^2^H_11/2_,^4^S_3/2_ → ^4^I_15/2_	0.0048 (467)	[48]
Er:Bi_5_TiNbWO_15_	175–550	^2^H_11/2_,^4^S_3/2_ → ^4^I_15/2_	0.0037 (385)	[49]
Er/Yb:NaLuF_4_	303–363	^2^H_11/2_,^4^S_3/2_ → ^4^I_15/2_	0.0031 (303)	[50]
Er/Yb:CaWO_4_	303–873	^4^G_11/2_,^2^H_9/2_ → ^4^I_15/2_	0.0073 (873)	[51]
Er/Yb:La_2_O_3_	303–600	^2^H_11/2_,^4^S_3/2_ → ^4^I_15/2_	0.0091 (303)	[52]
NaLuF_4_:Yb/Er/Tm	120–300	^2^H_11/2_,^4^S_3/2_ → ^4^I_15/2_	0.0019 (300)	[53]
LiYF_4_:Yb/Er/Ag	303–348	^2^H_11/2_,^4^S_3/2_ → ^4^I_15/2_	0.0095 (303)	This work

This may be linked to the highest sensitivity among other host materials, as displayed in Table 1. Furthermore, we observed that sensitivity of LiYF_4_:Yb^3+^/Er^3+^/Ag at 303 K is also higher than LiYF_4_:Yb^3+^/Er^3+^ manifested to a highly efficient photon to plasmon conversion of Ag nanoparticles in microfibers. The Ag co-doped microfibers are intrinsically immune to photobleaching which provided high stability dopant for optical sensing. It suggests that Ag co-doped fibers due to significant sensing properties are suitable for temperature recognition. As a result, the utilization of Ag nanoparticles in a microfiber is beneficial to increase the luminescence and to tailor thermal sensing properties, suggesting a promising sensitive temperature sensor.

## Conclusions

In summary, tetrogonal-LiYF_4_:Yb^3+^/Er^3+^ were prepared via thermal decomposition method and fibers were fabricated after co-doping PMMA solution with Ag and UCNPs. Successful Ag incorporation in UCNPs was supported through SEM, TEM, EDS, XPS, FTIR, and TGA analysis. The Ag co-doped polymer microfibers with a wave-guiding excitation approach and demonstrated potential use in thermal sensor were investigated. The intensity-dependent temperature sensitivity of Ag microfiber (0.0095 K^°1^) is higher than undoped Ag (0.0065 K^°1^) at 303 K, proposing Ag-doped microfibers are potential candidates for upgrading intensity-based temperature sensitivity at room temperature, which opens up new opportunities for developing compact photonic and plasmonic devices with low optical power. In the development of a newly employed method of microfibers with specified properties, significant improvements in up-conversion enhancement may be possible, leading to a more efficient up-converter, thereby enabling many of the technological applications of these materials.

## Data Availability

All data are fully available without restriction.

## Abbreviations

JCPDS: Joint committee on powder diffraction standards
CCD: Charge-coupled device
UCNPs-MF: Up-conversion nanoparticles microfibers
UC: Up-conversion
PL: Photoluminescence
Ln^3+^: Trivalent lanthanide ions
LiYF_4_:Er^3+^/Yb^3+^: 1%Er^3+^/22%Yb^3+^
LiYF_4_:Er^3+^/Yb^3+^/Ag: 1%Er^3+^/22%Yb^3+^/0.003g
RE: Rare earth ions
XRD: X-ray diffraction
TEM: Transmission electron microscope
SEM: Scanning electron microscope
EDS: Energy dispersive X-ray spectroscopy
XPS: X-ray photoelectron spectroscopy
FTIR: Fourier transform infrared rays
TGA: Thermal gravimetric analysis
FIR: Fluorescence intensity ratio
△E: Energy difference
SA: Absolute sensitivity
