# Correction to: Polymer Microfibers Incorporated with Silver Nanoparticles: a New Platform for Optical Sensing

**DOI:** 10.1186/s11671-019-3136-2

**Published:** 2019-09-04

**Authors:** Muhammad Khuram Shahzad, Yundong Zhang, Adil Raza, Muhammad Ikram, Kaiyue Qi, Muhammad Usman Khan, Muhammad Jehanzaib Aslam, Abdulaziz Alhazaa

**Affiliations:** 1 National Key Laboratory of Tunable Laser Technology, Institute of Opto-Electronics, Department of Electronic Science and Technology, Harbin Institute of Technology (HIT), Harbin, 150080 People’s Republic of China; 2 College of Material Science and Technology, Nanjing University of Aeronautics and Astronautics, 29 Yudao Street, Nanjing, 210016 People’s Republic of China; 3 Solar Cell Applications Research Lab, Department of Physics, Government College University, Lahore, Punjab 54000 Pakistan; 4 Research Chair for Tribology, Surface, and Interface Sciences, Department of Physics and Astronomy, College of Science, King Saud University, Riyadh, Saudi Arabia; 5 King Abdullah Institute for Nanotechnology, King Saud University, Riyadh, Saudi Arabia

**Correction to: Nanoscale Res Lett (2019) 14:270.**

**https://doi.org/10.1186/s11671-019-3108-6**

Please note that following publication of the original article [1], these three sentences have been removed from the Background section of the article:

1. “Heterostructure Cu2O/ZnO is a promising material structure which leads to a functional integration of the properties of Cu2O and ZnO also to novel interface effects and phenomena [7].”
2. “LiYF4:Yb3+/Er3+ were synthesized using thermal decomposition method and co-doped with Ag nanoparticles (NPs), and PMMA solution was exploited to fabricate microfibers.”
3. “Moreover, a single microfiber having thermal sensitivities using transitions 2 H11/2 → 4 I15/2 and 4 S3/2 → 4 I15/2 levels at 522 and 541 nm will be evaluated.”

The reason being, the authors flagged that these sentences (indirectly) duplicated content positioned immediately before or after the sentences (before for ‘1’ and ‘3’; after for ‘2’).
